# Low-Intensity, High-Frequency Grazing Positively Affects Defoliating Behavior, Nutrient Intake and Blood Indicators of Nutrition and Stress in Sheep

**DOI:** 10.3389/fvets.2021.631820

**Published:** 2021-06-22

**Authors:** Angel S. Zubieta, Alejandra Marín, Jean V. Savian, Anderson M. Soares Bolzan, Jusiane Rossetto, Mariana T. Barreto, Jéromê Bindelle, Carolina Bremm, Laura V. Quishpe, Stella de Faria Valle, Virginie Decruyenaere, Paulo C. de F. Carvalho

**Affiliations:** ^1^Grazing Ecology Research Group, Federal University of Rio Grande Do Sul, Porto Alegre, Brazil; ^2^Facultad de Ciencias Agrarias, Departamento de Producción Animal, Universidad Nacional de Colombia, Medellín, Colombia; ^3^Instituto Nacional de Investigación Agropecuaria, Programa Pasturas y Forrajes, Estación Experimental INIA Treinta y Tres, Treinta y Tres, Uruguay; ^4^Animal Production and Ruminant Nutrition Research Group, Federal University of Pampa, Rua Promorar Luiz Joaquim de Sá Brito, Itaquí, Brazil; ^5^Precision Livestock and Nutrition Unit, Gembloux Agro-Bio Tech, TERRA, Teaching and Research Centre, University of Liège, Gembloux, Belgium; ^6^Department of Agricultural Research and Diagnosis, Secretariat of Agriculture, Livestock and Rural Development, Porto Alegre, Brazil; ^7^Department of Clinical Veterinary Pathology, Federal University of Rio Grande Do Sul, Porto Alegre, Brazil; ^8^Productions in Agriculture Department, Animal Production Unit, Walloon Agricultural Research Centre (CRA-W), Gembloux, Belgium

**Keywords:** animal welfare, nutritional status, grazing management models, sward height, ingestive comfort

## Abstract

The intensity and frequency of grazing affect the defoliating strategy of ruminants, their daily nutrient intake, thus nutrition and physiological status. Italian ryegrass (*Lolium multiflorum* Lam.) pastures were grazed by sheep either under a low-intensity/high-frequency grazing strategy (Rotatinuous stocking; RN) with nominal pre- and post-grazing sward heights of 18 and 11 cm, respectively, or under a high-intensity/low-frequency strategy (traditional rotational stocking; RT) with nominal pre- and post-grazing sward heights of 25 and 5 cm, respectively. Treatments were arranged under a complete randomized design and evaluated over two periods, in different years. In 2017, the aim was to depict the type of bites that sheep perform during the grazing-down and associate them to the grazing management strategy according to their relative contribution to the diet ingested. In 2018 we estimated the total nutrient intake and evaluated blood indicators of the nutritional status and immune response to stress of sheep. The bite types accounting the most for the diet ingested by RN sheep were those performed on the “top stratum” of plants with around 20, 15, and 25 cm, whereas the type of bites accounting the most for the diet of RT sheep were those performed on “grazed plants” with around 10, 5, and ≤ 3 cm. In 2018, the RN sheep increased by 18% the total organic matter (OM) intake and by 20–25% the intake of soluble nutrients (i.e., crude protein, total soluble sugars, crude fat), digestible OM and of metabolizable energy, and had 17.5, 18, and 6.1% greater blood concentration of glucose, urea nitrogen (BUN) and albumin, respectively, but 17% lower blood neutrophil-to-lymphocyte (N:L) ratio. Sheep grazing vegetative Italian ryegrass pastures under the low-intensity/high-frequency grazing strategy (RN) ingested a diet of better quality from bites allocated on the top stratum of plants, had greater intake of soluble nutrients and blood parameters positively associated with nutritional status and immune response to stress.

## Introduction

Criteria used to define the limits of sward depletion affect the foraging strategy of ruminants, thus the herbage intake and diet quality. Carvalho (1) proposed a low-intensity/high-frequency grazing approach that defines the management limits of the sward based on animal behavioral responses, i.e., a pre-grazing sward height allowing animals to maximize the intake rate and a grazing-down of 40% to sustain it high at any time while grazing. Although maximizing the intake rate is a natural foraging strategy of ungulates (2), applying this grazing approach is non-sense in most commercial farms, as orientations to maximize intake rate and individual animal intake are thought to reduce harvest efficiency and farm profit (3, 4). Conversely, for increasing herd forage intake and making full exploitation of the area, traditional guidelines propose starting grazing when the balance between herbage accumulation and its quality is optimized (5), and low residual sward height or mass as depletion limit (6–8), through high-intensity/low-frequency grazing strategies (9).

High-intensity/low-frequency grazing force animals to extend sward depletion to bottom parts of plants, preventing them from allocating bites on leaf laminas of the top stratum, which restrict the individual intake of a more digrestible diet (1). The metabolic profile of animals is directly affected by the intake of digestible organic matter [OM; (10)]. For instance, when blood glucose is low, other products coming from lipolysis of body reserves become available [e.g., non-esterified fatty acids (NEFA), beta-hydroxybutyrate (BHB)], affecting the proliferation of immune cells [e.g., leukocytes; (11, 12)]. Therefore, apart from being against the natural preference for leaf laminas and of ingestive comfort associated with high and profitable intake rates (13), lower daily intake of a less digestible diet could threaten some of the domains of animal welfare [e.g., nutritional status and immune response; (14)], even when pastoral systems claim to promote it (15).

If the defoliating behavior of non-supplemented grazing animals affects their physiology, pastoral systems could improve or impair their nutrition and welfare depending on grazing management. We hypothesized that sheep grazing Italian ryegrass (*Lolium multiflorum* Lam.) pastures under a low-intensity/high-frequency grazing strategy and composing a diet from bites performed preferentially on the top stratum of plants, have greater intake of a diet with better quality and blood parameters positively associated with nutritional status and immune response to stress, compared to animals under a high-intensity/low-frequency traditional management (RT), composing a diet with lower quality from bites performed on both top and grazed parts of plants. To test this assumption, we conducted two grazing trials over two consecutive years. In 2017, we characterized, at the bite scale, the diet ingested by sheep grazing Italian ryegrass pastures, and in 2018, we determined the daily nutrient intake and compared the impact of the grazing management strategy on nutrition- and stress-related blood parameters of sheep.

## Materials and Methods

All procedures on animals followed the guidelines of the law of procedure for the scientific handling of experimental animals and were approved by the Ethics Committee for the Use of Animals (CEUA) of the Federal University of Rio Grande do Sul (UFRGS; protocol 3571).

### Experimental Area and Pasture Establishment

The grazing trials were conducted at the Experimental Station of the Faculty of Agronomy of the UFRGS, in Southern Brazil (30°05”22' S latitude, 51°39”09'W longitude and 46 m above sea level), with a subtropical humid “Cfa” climate with an average annual temperature of 18°C. Italian ryegrass pastures were established on April 20th in 2017 and May 23th in 2018, through conventional soil preparation (plowing and disking), mechanical spreading of 35 kg of seed per hectare and 250 kg of the formula (NPK, 5-30-15) per hectare at seeding and 200 kg of nitrogen (urea) 30 days later.

### Treatments and Experimental Design

Two grazing management strategies in rotational stocking were evaluated under a completely randomized design, with two paddocks per treatment in 2017 and with four in 2018, over two evaluation periods. The Rotatinuous stoking (RN), with nominal pre- and post-grazing heights of 18 and 11 cm, respectively, was compared with a traditional rotational stocking (RT), with nominal pre- and post-grazing heights of 25 and 5 cm, respectively. The combination of pre- and post-grazing sward heights results either in low-intensity/high-frequency (RN) or in high-intensity/low-frequency (RT) grazing strategies (9). For the RN, the pre-grazing height aims to maximize intake per unit of grazing time on Italian ryegrass pastures, while the post-grazing height (40% reduction of the initial height) aims to sustain the intake rate at any time while grazing (1, 16, 17). For the RT, the pre-grazing height aims to initiate grazing at maximum net herbage accumulation and the post-grazing height to maximize herbage harvest efficiency (9, 18).

### Animals and Pasture Management

The number of animals, paddocks and the duration of the stocking period differed between 2017 and 2018, according to year-specific objectives. Twelve Texel sheep (35 ± 4.3 kg of live weight; LW) in 2017, and 24 Texel × Corriedale castrated males (41.1 ± 3.4 kg LW) in 2018, were randomly allocated, respectively, to four and eight paddocks of 0.21 ha (three test animals per paddock). Pasture management was similar in both years. Briefly, sheep grazed in strips, changing to another daily between 14:00 and 15:00 h. Thereby, each paddock was subdivided into strips of variable size (130 and 47 m^2^ on average for RN and RT, respectively), according to treatment targets and herbage growth; strips provided enough forage for animals to deplete the sward within the preestablished pre- and post-grazing sward heights.

The pre- and post-grazing sward heights were measured at two-day interval during the treatment adaptation period and daily during evaluations, by taking 150 random readings per strip at the “leaf horizon” on the top of the sward, with the aid of a sward stick (19). To maintain sward height targets, a variable number of put-and-take sheep, accompanied the three test-sheep on each strip (20). Sheep entered to paddocks before the pre-grazing sward height of both treatments was achieved, to complete a grazing cycle on all paddocks, while creating a sward height gradient. This allowed the first grazed strip within each paddock to reach the treatment pre-grazing sward height, just before it was grazed again. Thereby, from the second grazing cycle onwards on paddocks of each treatment, animals grazed on regrown sward strips within preestablished treatments heights; at this moment, the period of adaptation to treatments of 35 days in 2017 and of 16 days in 2018, started. Afterwards, in both 2017 and 2018, two evaluation periods took place on vegetative swards. Animals always had free access to water.

### The Continuous Bite-Monitoring and Bite-Scale Hand-Plucking

**Figure 1:**
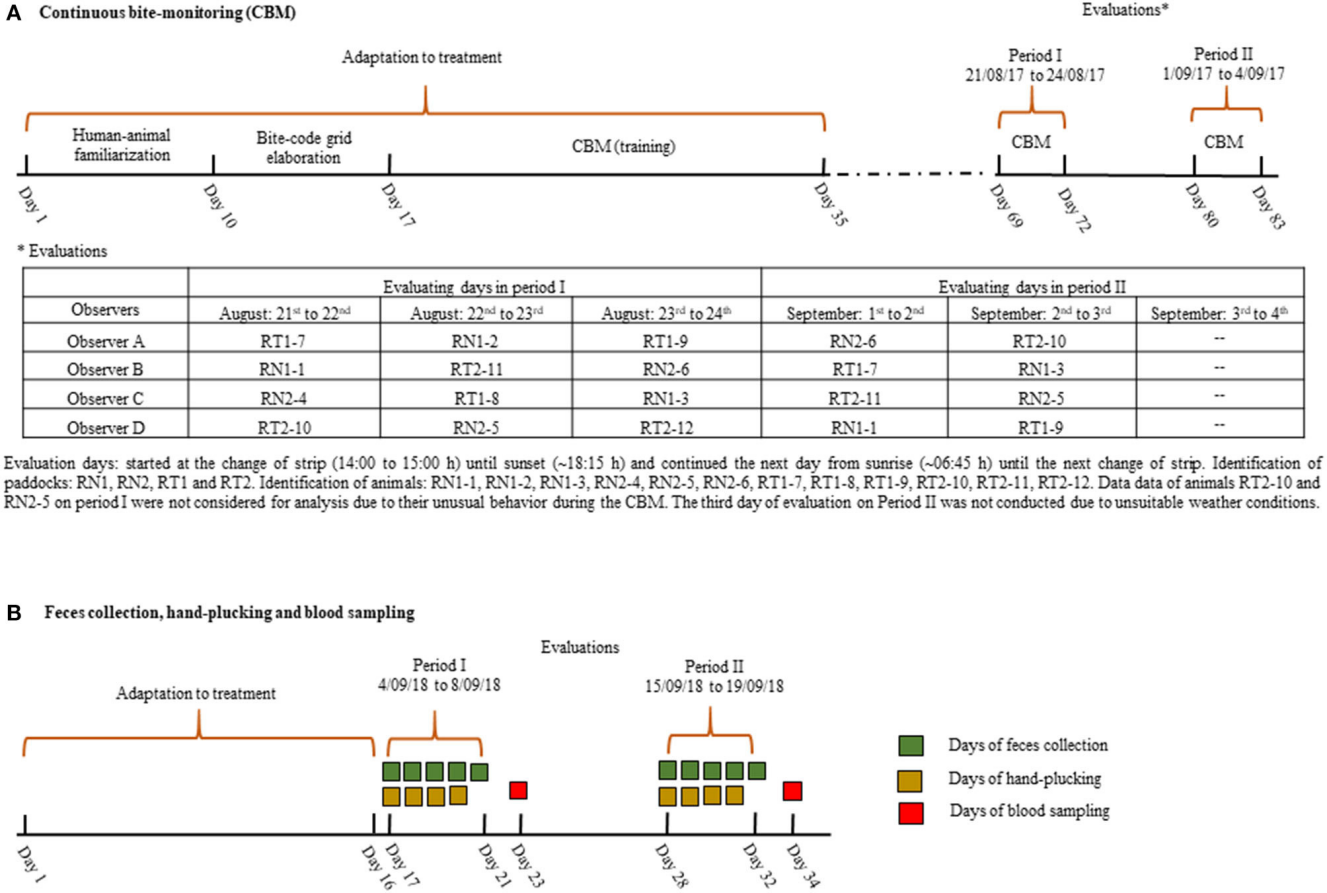
Experimental scheme during the continuous bite-monitoring (CBM) in 2017 **(A)**, and feces collection, hand-plucking and blood sampling in 2018 **(B)**.

**Figure 2:**
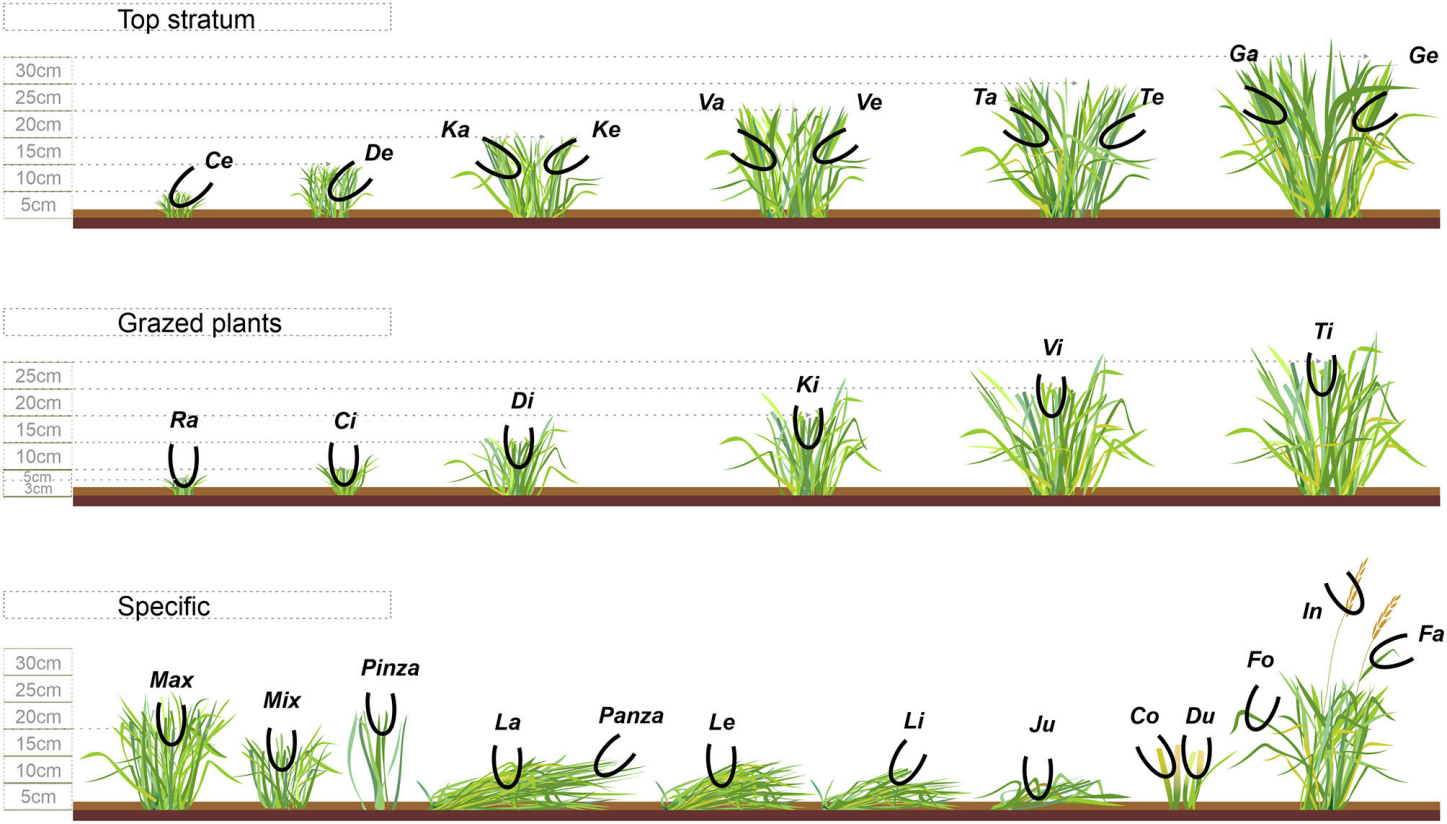
Bite-code grid of sheep grazing vegetative Italian ryegrass pastures under contrasting grazing management strategies (RN and RT) in rotational stocking. The pictogram illustrates the bite types classified into three categories: top stratum, grazed plants or specific. Among the bites performed on “top stratum” are those coded with the consonants “C, D, K, V, T and G,” indicating plants of around 5, 10, 15, 20, 25 and 30 cm (± 2.5 cm), respectively; yet, the vowels “a” and “e” after these consonants indicate, respectively, “dense” and “less dense” bites (≥4 or ≤3 intact leaf laminas, respectively). Among the bites performed on “grazed plants” are those coded with the vowel “i” after the consonants, which also indicates leaf laminas with presence or not of stems; the bite “Ra,” within this category, is performed on plants of ≤3 cm with minimum or no presence of leaf laminas. Among the “specific” type of bites, the bite “Max” is allocated on both grazed and intact leaf laminas, with the presence or not of stem, on plants with ≥15 cm; the bite “Mix” is similar, but on plants with ≤15 cm; the bite “Pinza” is allocated at the tip (superficial) of a single or no more than two intact or grazed leaf laminas, and at any sward height; the bite “Panza” is also a superficial bite, but on lying plants; the bite “La” is allocated on 2-to-3 lying intact leaf laminas with ≥20 cm length, while the bite “Le” is similar, but on plants with ≤20 cm length; the bite “Li” is allocated on lying plants gathering 2–3 grazed leaf laminas of any horizontal length; the bite “Co” and “Du” are performed on one and two defoliated stems, respectively, on plants with 5–15 cm; the bite “Fo” is performed on a single intact or grazed leaf lamina within the canopy; the bite “Fa” is performed on the leaf lamina below the inflorescence, intact or grazed; the bite “In” is allocated on the inflorescence; the bite “Ju” is allocated on a trampled plant, in which animals manipulate and gather 2–4 intact or grazed leaf laminas.

In 2017, we implemented the continuous bite-monitoring (CBM) methodology consisting of (1) animal-observer familiarization, (2) bite-code grid elaboration, (3) observer training the bite-code grid monitoring and (4) real-time bite-monitoring evaluation [for details see (21–23)]. We used this method during two periods, with 3 days of observation, as schematized in Figure 1A. Briefly, during the first 10 days of adaptation to the treatment, four observers accustomed animals to the proximity of humans (<1 m) and during the following 25 days, the observers trained the bite-coding grid (Figure 2) previously elaborated for sheep grazing vegetative Italian ryegrass swards, and only when they were able to encode, in real time, each bite without hesitation, observations initiated. Each observer evaluated a different animal each day, alternating the treatment each day. Despite precautions, in period one, a sheep of each treatment had to be discarded from the analysis as they presented unusual behavior in the presence of the observer. Indeed, in period two, the 3rd day of observation was not conducted due to unsuitable weather conditions; thus, only two out of the three test animals within a paddock were evaluated during two observation days. Overall, 18 out of 24 possible observations were obtained. The bite encoding was recorded with a digital Sony recorder Icd-PX240^®^ device. Recordings of the bite-monitoring were analyzed using the software JWatcher^®^ (http://www.jwatcher.ucla.edu/, verified 10 December 2019; The Observer, Noldus Information Technology^®^, The Netherlands). While animals were not performing any eating activity during the CBM, the observers simulated at least 20 times each bite type (Figure 2), as detailed in Bonnet et al. (24); fresh samples were put on a paper bag. The total dry mass (g DM) of each bite type was calculated by oven drying the mass gathered per bite, at 55°C for 72, and dividing the dry weight on an electronic scale (0.0001 g precision) over the number of simulations; this information was used to compose the diet ingested.

#### Calculation of the Dry Matter Intake per Bite Type (Diet Ingested)

To determine the sheep DM intake per bite type, we multiplied the number of times that each bite type was recorded during the CBM by their individual dry mass (g DM). Afterwards, we summed up the DM ingested by all the bite types to estimate the intake during the CBM. Finally, the DM intake per bite type was divided by the intake of each animal to obtain the relative proportion that each bite type accounted for in the DM intake; herein referred as the diet ingested.

### Total Organic Matter Intake and Herbage Digestibility

In 2018, the daily OM intake was estimated on the three test-sheep per paddock in two periods (Figure 1B). We used the fecal crude protein (CP) technique (25), as described by Savian et al. (18). The equation proposed by Azevedo et al. (26) for Italian ryegrass was used: OM intake = 111.33 + 18.33 × fecal CP (g/sheep/day). Each period consisted in total feces collection during five consecutive days. Sheep were fitted with feces collecting bags, which were emptied once per day (from 07:00 to 08:30 h) and the feces were weighed and homogenized to take sub-samples of 20% of the total. Fecal samples were dried at 55°C for 72 h, pooled per sheep, grounded and stored until analysis; afterwards, they were put on an oven at 550°C for 4 h to obtain the ash content. The OM content was calculated by subtracting the ash content from the dried mass of samples. The nitrogen content was obtained by the Kjeldahl method (27), and the CP content was calculated by multiplying the nitrogen content by 6.25. The OM digestibility was calculated as follows: OM digestibility = 1–total amount of feces (g DM/sheep/day)/OM intake (g DM/sheep/day). The digestible OM intake was calculated using the OM intake and OM digestibility. The metabolizable energy (ME) intake was estimated using the model proposed by CSIRO, [(28); ME = 0.169 × OM digestibility−1.986].

### Nutrient Content of the Diet Ingested and Total Daily Nutrient Intake

In 2018, each bite of the bite-code grid (Figure 2) was hand-plucked, as described above, during the first 4 days of the feces collection (Figure 1B). The simulation was performed by one evaluator after the main morning and afternoon meal bouts, completing one paddock per treatment per day; all bite types were simulated once on each paddock on each period. The fresh mass of bites was put on a cooler with ice immediately after sampling, and within 4 h stored at −20°C until freeze-drying (freeze-dryer Martin Christ DELTA 1-24 LSC, Germany) and grounding (1 mm screen). From these bite samples, we compounded 16 diets of 10 g of lyophilized samples (2 treatments × 4 paddocks × 2 periods), considering the proportion that each bite type accounted for to the diet ingested, as estimated in 2017. The herbage nutrient contents (g kg/DM) of diets were estimated by NIRS scanning (XDS NIRS system, FOSS—Denmark, 1,100–2,498 nm of wavelength by 2 nm steps and absorbency data expressed as log 1/R), using calibrations developed at the Walloon Agricultural Research Center (CRAW), Belgium (29). The daily OM intake estimated by the fecal CP technique was converted to DM intake (g/animal/day) by dividing the daily OM intake over the OM content of the diet, derived from NIRS. From this, we calculated the daily nutrient intake, as follows: daily nutrient intake = total DM intake (g/animal/day) × nutrient content (g/kg DM).

### Biochemical and Hematological Blood Parameters

In 2018, sheep blood samples (4 mL) from the jugular vein were collected on tubes containing heparin (EDTA K2) and on non-heparinized tubes (Inforlab, São Paulo, Brazil), 2 days after the last fecal collection day of each period from 07:00 to 08:30 h (Figure 1B). Samples were transported in a cooler at 5°C, within 4–6 h, to the Department of Clinical Veterinary Pathology of the UFRGS. Packed cell volume was assessed from heparinized samples with the micro hematocrit method; samples (duplicate) were diluted with Turks's solution and centrifuged (Heraeus Pico 17, Thermo Scientific) on capillary tubes at 17,000 *g* for 5 min at room temperature (17°C) to perform total leukocyte counting (hemocytometer Neubauer Improved, New Optics). Blood smears were dried and stained with Diff Quick to perform differential leukocyte counting (i.e., total, neutrophils, lymphocytes and blood neutrophil-to-lymphocyte ratio; N:L). Non-heparinized samples were brought to room temperature and centrifuged (Heraeus Megafuge 8, Thermo Scientific) at 1,700 *g* for 10 min. The serum obtained was analyzed for glucose, urea, albumin, alkaline phosphatase, fructosamine and cholesterol by enzymatic colorimetric analysis using commercial kits (Glucose HK, Urea Color 2R, Albumin AA, ALP 405 AA, Fructosamine AA and Colestat enzymatic, respectively, Wiener Lab., Rosario, Argentina) in a Wiener Lab CM 200 auto-analyzer (Wiener Lab., Rosario, Argentina). Considering that the molecular weight of urea is 2.14 times that of urea nitrogen, the blood urea nitrogen (BUN) was estimated from serum urea as follow: BUN (mg/dL) = serum urea (mg/dL)/2.14. An aliquot of centrifuged serum was stored in Eppendorf tubes (1.5 mL) and frozen at −20°C until analyzed separately by enzymatic colorimetric analysis for NEFA (Randox, Antrim, UK) and BHB (Ranbut, Randox, Antrim, UK).

### Statistical Analysis

Pasture data in 2017 and 2018 was subjected to ANOVA, at 5% of significance, considering the fixed effect of treatment, period and year, and their interactions, and the random effect of the paddock (experimental unit). The diet ingested by animals, estimated in 2017 from the CBM, was subjected to a multivariate analysis of variance (MANOVA), at 5% of significance, to compare the relative proportion that each bite type accounted to the diet ingested between grazing management (RN and RT), considering the fixed effect of treatment and period, and their interaction. Moreover, a principal component analysis (PCA) was performed to order the types of bytes performed by sheep grazing Italian ryegrass pastures under both grazing management strategies, according to their relative contribution to the diet ingested. The percent of the variance explained per axis was used as a selection criterion. Data of 2018, describing the nutrient content of the diet, the daily nutrient intake, OM digestibility and metabolizable energy content and blood parameters, was subjected to ANOVA at 5% of significance. The model included the fixed effects of the treatment, the random effects of the period and of the animal nested within the paddock (experimental unit), and of the treatment × period interaction (lmer function), except for the data of the nutrient content of the diet, whose model excluded the effect of the animal nested within the paddock. The statistical models in both years were selected considering the best fit model according to the AICs' criteria. Means were compared using the least-squares mean linear hypothesis test adjusted for Tuckey comparison. All analyzes were performed using the R software [(30), version 3.6.0].

## Results

### Sward Canopy Height and Bite-Scale Characterization of the Diet Ingested

**Figure 3:**
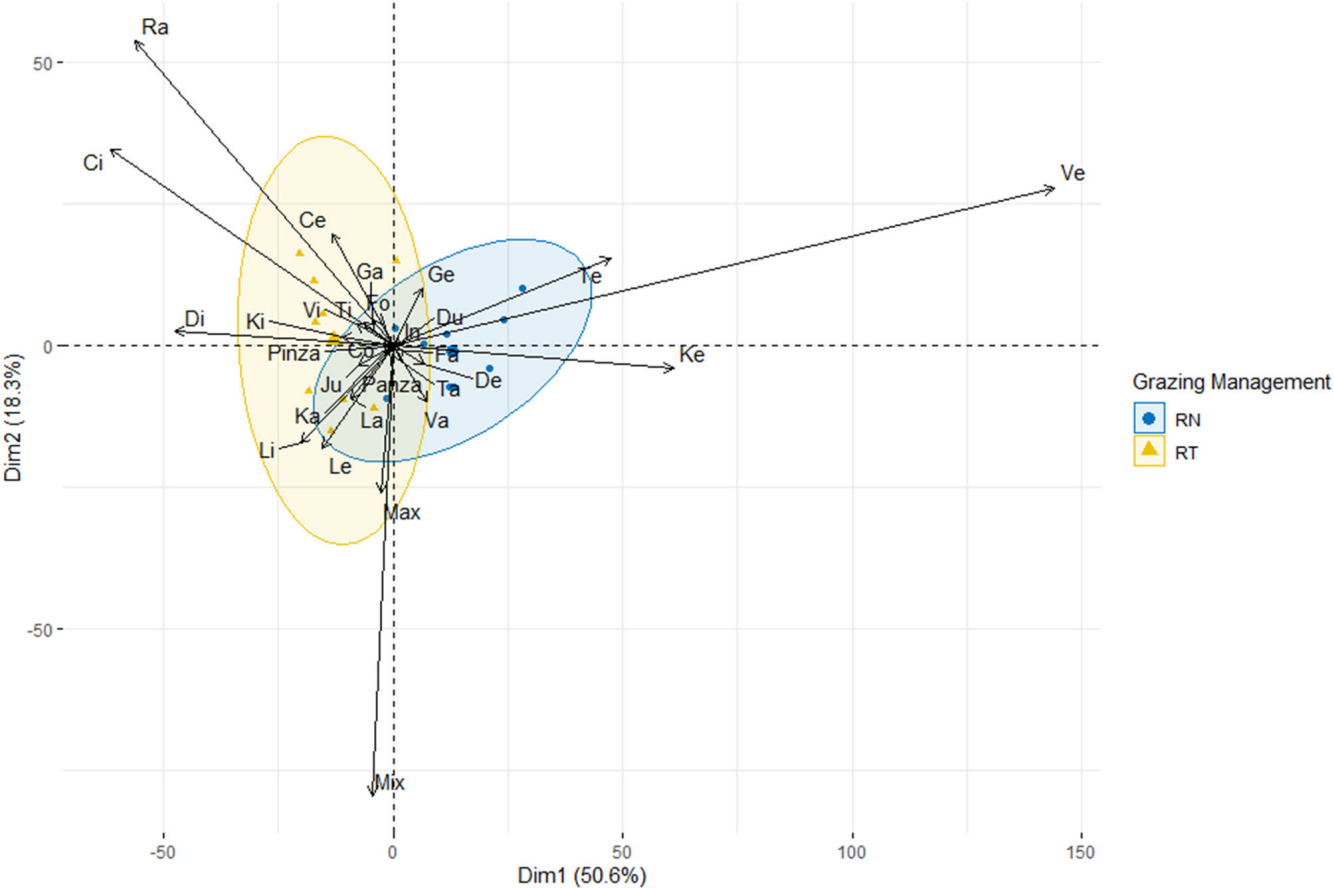
Principal Component Analysis (PCA). Ordination diagram of the types of bytes performed by sheep grazing vegetative Italian ryegrass pastures under different grazing management strategies (RN: blue circles, RT: yellow triangles), according to their relative contribution on the diet ingested.

Table 1 shows the difference in pre- and post-grazing sward heights between treatments. The effects of year and period, and their interaction with treatments did not affect the pre- and post-grazing sward canopy height or the sward height depletion (*P* > 0.05). The management of the sward height affected the proportion that some, but not all bite types, accounted to the diet ingested (*P* < 0.001; Table 2). Neither the effect of the period nor its interaction with treatment was significant. Figure 3 shows the first two dimensions of the PCA, explaining 69% of the total variation of data. The bite *Ve*, performed on the top stratum of plants with ~20 cm, was the most associated with the RN management, followed by the bite *Te* and *Ke*, performed also at the top stratum of plants with ~25 and ~15 cm, respectively; these three bite types accounted for 47 and 15% of the diet ingested by the RN and RT sheep, respectively. Overall, the bites of the top stratum of plants accounted for 60 and 27% of the diet ingested, respectively, by the RN and RT sheep (Table 2). Bites that associated the most with the RT management were *Ci, Di* and *Ra*, performed on grazed plants with ~5, ~10, and ≤3 cm, respectively; these three bite types accounted for 11 and 35% of the diet ingested, respectively, by the RN and RT sheep. Overall, these three and the other bite types performed on grazed plants accounted for 22 and 47% of the diet ingested, respectively, by the RN and RT sheep (Table 2). Other specific type of bites whose contribution in the diet differed between treatments, were those performed on lying plants, trampled plants or steams, namely *La, Li, Le, Jun*, and *Co*, and accounted for 1 and 11% of the diet ingested by the RN and RT sheep, respectively; the contribution to the ingested diet of some of the bite types performed on the top stratum, grazed plants or specific, did not differ between the RN and RT strategies (Table 2).

**Table 1:** Sward surface height and sward height depletion of vegetative Italian ryegrass pastures grazed by sheep under contrasting grazing management strategies (RN and RT) in rotational stocking.

**Variables**	**RN**	**RT**	**SEM**	***P-value***
Pre-grazing, cm	19.4	27.3	0.4	<0.0001
Post-grazing, cm	12.2	6.9	0.3	<0.0001
Sward height depletion, %	37.3	74.7	1.8	<0.0001

*RN, Rotatinuous stocking; RT, Traditional rotational stocking; SEM, standard error of the mean*.

**Table 2:** Bite-scale characterization of the diet ingested by sheep (relative proportion that each bite type accounted to the diet ingested) grazing vegetative Italian ryegrass pastures under contrasting grazing management strategies (RN and RT) in rotational stocking.

**Bite type**	**RN**	**RT**	**SEM**	***P*-Value**
**Top stratum (less dense)**				
Ce	0.69	3.48	0.87	0.134
De	3.30	2.18	0.35	0.092
Ke	11.19	5.23	1.52	0.031
Ve	24.07	5.76	2.88	0.000
Ge	3.54	2.25	0.77	0.472
Te	11.3	3.67	1.31	0.001
**Top stratum (dense)**				
Ka	0.65	0.45	0.24	0.768
Ta	1.51	1.16	0.28	0.425
Va	2.81	0.96	0.50	0.062
Ga	0.66	1.47	0.30	0.188
**Grazed plants**				
Ra	0.40	9.24	1.73	0.004
Ci	3.44	12.66	1.48	0.000
Di	7.23	13.34	1.22	0.008
Ki	5.93	6.70	1.01	0.698
Vi	3.61	3.19	0.69	0.824
Ti	0.99	1.425	0.42	0.585
**Specific**				
Mix	10.77	10.77	1.64	0.983
Max	6.30	4.215	0.85	0.271
Pinza	0.35	0.275	0.10	0.256
Panza	0	0.16	0.06	0.736
La	0	1.68	0.37	0.020
Le	0.57	3.53	0.69	0.027
Li	0.13	3.86	0.66	0.001
Jun	0	1.565	0.30	0.004
Co	0.01	0.305	0.05	0.000
Du	0	0.085	0.02	0.059
Fa	0.21	0.215	0.04	0.981
Fo	0.08	0.135	0.03	0.296
In	0.01	0.005	0.005	0.391

*RN, Rotatinuous stocking; RT, Traditional rotational stocking. SEM, standard error of the mean*.

### Nutrient Content of the Diet Ingested and Daily Nutrient Intake

Table 3 shows the effect of treatments on the nutrient content of the diet ingested and on total daily nutrient intake; neither the effect of the period nor its interaction with treatment was significant (*P* > 0.05). In the RN management, the CP and total soluble sugar contents of the diet ingested were greater (*P* < 0.001), the crude fat content tended to be greater (*P* = 0.056), while the fibrous compounds such as ADF and ADL contents were lower (*P* < 0.001), with no difference for NDF content between treatments (*P* > 0.05). OM digestibility and ME content greater in the RN diet (*P* < 0.0001). The intake of OM, digestible OM, and all nutrients, except ADF (*P* > 0.05), were greater for the RN management (*P* < 0.001).

**Table 3:** Nutrient content of the ingested diet and total nutrient intake by sheep grazing vegetative Italian ryegrass pastures under contrasting grazing management strategies (RN and RT) in rotational stocking.

**Variable**	**RN**	**RT**	**SEM**	***P-value***
**Nutrient content, g/kg DM**				
Organic matter (OM)^a^	896	883	3.4	0.036
Crude protein (CP)^a^	254	220	5.5	<0.0001
Neutral detergent fiber (NDF)^a^	358	367	9.7	0.4086
Acid detergent fiber (ADF)^a^	259	321	13.9	0.009
Acid detergent lignin (ADL)^a^	23	33	2.7	0.027
Total soluble sugars^a^	144	126	3.4	0.0086
Crude fat^a^	41	37	1.9	0.056
OM digestibility, g/kg OM	771	755	2.2	<0.0001
Metabolizable energy, MJ/kg DM	11.05	10.77	0.04	<0.0001
**Daily intake, g/animal**				
Organic matter (OM)	835.95	680.90	54.3	<0.0001
Crude protein (CP)	237.1	169.5	7.2	<0.0001
Neutral detergent fiber (NDF)	333.8	281.6	8.9	<0.0001
Acid detergent fiber (ADF)	241.7	248.4	7.4	0.586
Acid detergent lignin (ADL)	21.5	25.3	1.1	<0.0001
Total soluble sugars	135.2	96.9	4.0	<0.0001
Crude fat	38.1	28.6	1.2	<0.0001
Digestible OM	643.5	513.6	16.9	<0.0001
Metabolizable energy, MJ/day	10.3	8.3	0.3	<0.0001

*RN, Rotatinuous stocking; RT, Traditional rotational stocking. SEM, standard error of the mean*.

a *Values estimated by NIRS*.

### Blood Biochemistry and Hematology

Table 4 shows blood biochemical and hematological parameters of sheep as affected by the grazing management strategy. Neither the effect of the period, nor its interaction with treatment affected blood variables (*P* > 0.05). The serum concentration of albumin, glucose and urea (BUN) were greater in the RN sheep (*P* < 0.011), while the NEFA tended to be greater on RT animals (*P* = 0.09), and the alkaline phosphatase, fructosamine, cholesterol and BHB were unaffected by treatments (*P* > 0.05). The hematology profile shows lower lymphocyte counting (*P* = 0.05) and greater neutrophil-to-lymphocyte ratio (N:L; *P* < 0.046) on the RT sheep, and unaffected total leukocytes and neutrophil number, and neutrohpils and lymphocytes percentages (*P* > 0.05).

**Table 4:** Biochemical and hematological parameters of sheep grazing vegetative Italian ryegrass pastures under contrasting grazing management strategies (RN and RT) in rotational stocking.

**Variables**	**RN**	**RT**	**SEM**	**Reference values**	***P-value***
**Biochemical**					
Albumin (g/dL)	3.76	3.53	0.04	2.4–3.0^a^	0.011
Alkaline phosphatase (U/L)	260.1	231.4	10.8	68–387^a^	0.335
Fructosamine (umol/L)	254.3	233.3	4.5	–	0.175
Glucose (mg/dL)	61.0	50.4	1.3	50–80^a^	0.001
BUN (mg/dL)	30.8	25.3	0.7	8–20^a^	0.003
Cholesterol (mg/dL)	75.6	84.5	2.4	52–76^a^	0.478
BHB (mmol/L)	0.327	0.309	0.02	0.55^a^	0.170
NEFA (mmol/L)	0.12	0.24	0.02	–	0.092
**Hematological**					
Total leukocytes (cells/μL)	6853.0	6231.1	166.7	4,000–8,000^b^	0.11
Neutrophils (cells/μL)	1183.6	1272.8	71.3	700–6,000^b^	0.76
Lymphocytes (cells/μL)	3755.8	3358.7	127.7	2,000–9,000^b^	0.05
Neutrophils (%)	17.46	20.44	1.1	10–50^b^	0.133
Lymphocytes (%)	55.3	53.9	1.4	40–55^b^	0.109
N:L	0.315	0.379	0.03	–	0.046

*RN, Rotatinuous stocking; RT, Traditional rotational stocking*.

a *Reference value from Kaneko et al. (31) or*

b *Byers and Kramer (32). SEM, standard error of the mean*.

## Discussion

The RN stocking stresses the central role that the sward height has over the intake rate and daily forage intake of grazing ruminants. Boval and Sauvant (13) mentioned that foraging decisions driving the intake rate provide information of ingestive comfort, appetite, gut health and welfare. Indeed, Mellor (14) suggests that eating enough of a high-quality diet and experiencing postprandial satiety are components of a good mental and nutrition state, thus of welfare. Thereby, the RN stands as a grazing management that might promote welfare, as it mimics the “time minimizing” nature of grazers by allowing them to graze at high intake rates and to “take the best and leave the rest” of plants (1). We show this by finely depicting the diversity of bites that sheep can perform to cope with contrasting grazing strategies and compose their diet, and the effect that the resulting amount and quality of the diet ingested have over some nutrition- and stress-related blood parameters of sheep grazing vegetative Italian ryegrass pastures.

### The Biting Behavior and Nutrient Content of the Diet Ingested

Bergman et al. (2) suggest that ungulates are “time minimizers” or the opposite, intake rate maximizers, this is, that they opt to graze on sites allowing them to harvest the greater amount of food in the least possible time. This could be a foraging strategy adopted by grazers for fitness (i.e., surviving until reproductive age, finding a mate and producing an offspring). From this assumption, our observation that RN sheep composed their diet preferentially from bites performed on the top stratum of plants (i.e., leaf laminas), especially on Italian ryegrass plants with ~20 cm (Table 2) was expected, as around this sward height sheep display high intake rate on vegetative Italian ryegrass pastures (1), and also by assuming that herbivores defoliate swards from the top to the bottom of the canopy (33). Moreover, animals select leaves with greater soluble content (34) when they are not forced to explore the bottom parts of plants. As pointed out by Savian et al. (18), we also observed 14% greater CP content in the RN diet, with values within the range reported for ryegrass pastures grazed by sheep (35, 36). The 12% greater content of soluble sugars in the RN diet, but similar NDF content in both treatments, probably resulted from the low fiber content of the vegetative stage of Italian ryegrass in this study. The 2.2 and 2.5% greater OM digestibility and ME intake, respectively, by the RN animals (Table 3) could result in greater LW gain (9, 35) or milk yield (3). The 13% increase in crude fat was expected, with values within the range of other grazing trials with ryegrass (37–39). Several experiments showing the vertical quality gradient of forages (40–42) support our results indicating better nutritive value of the RN diet in response to preferential biting behavior on the top stratum of plants.

### Daily Nutrient Intake

According to Dove (43), as farm profit derives from outputs per hectare, balancing nutrient supply with demands should not be attempted on an individual-animal basis, instead, a daily penalization of ~10% of individual DM intake should be targeted (3); in dairy systems where the herbage utilization reaches 93% (6) restriction could arguably surpass 10%. Nonetheless, we suggest that optimizing individual nutrient intake from grazing would occur without the trade-off of reducing per hectare harvest efficiency and farm profit, provided that both primary (i.e., total herbage production and harvesting per stocking season) and secondary production (i.e., individual and per hectare LW gains), are both optimized with the RN stocking, with respect a RT applied on Italian ryegrass grazed by sheep (9). While setting sward heights for maximizing the intake rate does not mean that animals will always defoliate plants at such sward height or warrant maximum daily intake, the conditions are ideal for this to happen (1, 16, 17, 44), thus for reducing the supplementation with high-grain diets or silages. This is timely for high yielding animals [e.g., cows whose forage intake capacity increases by 0.18 kg/kg of milk at lactation peak on good quality pastures; (45)], under time-limiting scenarios. Clearly, the daily competence of grazing with other time-consuming behaviors (i.e., ruminating, idling, socializing, walking), human interventions (i.e., nocturnal housing, milking) or weather conditions (i.e., rain and fouling, heat stress, low forage growth), could reduce eating time and accentuate intake restrictions under scenarios of low intake rate (1).

### Blood Biochemistry and Hematology

The management of grazing affects the amount and quality of nutrients that animals ingest, thus their metabolic status. Blood glucose is a short-term proxy of energetic metabolism (46, 47), and in ruminants, propionic acid is its main precursor, although amino acids make a minor contribution to gluconeogenesis (48). Therefore, its greater concentration on RN animals could be explained by their greater intake of digestible OM and CP (Table 4). In line with this, Raja et al. (10) suggested that glucose responds positively to digestible OM intake. Glucose concentration in both RN and RT sheep (Table 4) is within reference values of adult sheep (31), and values in RN animals compare well with those of sheep grazing ryegrass at low intensity (49). Moreover, other reports coincide with this study in that animals with restricted feed intake, thus with limited supply of gluconeogenic substrates, have lower blood glucose concentration than better feed animals (50–53).

Fructosamine is formed from glucose and mainly albumin and owing to albumin's half-life of around 2 weeks (54), it serves as a blood marker of glycemia of the previous weeks. The non-significant increase in fructosamine in RN animals, despite higher blood glucose and albumin, could indicate the non-sensitivity of this proxy to acute changes of glucose (55), as observed on cows with less energy deficit after 30 days in lactation (56), or that a single glucose sampling did not allow an accurate referencing of glycemia of the previous weeks, because of day-to-day variation in DM intake in grazing conditions. Nonetheless, the reduction by half in blood NEFA concentration (*P* = 0.09) in RN sheep, is consistent with a better energy balance compared to RT sheep.

In this latter regard, energy intake restriction reduces cholesterol levels (57), triggers the mobilization of fatty acids from adipocytes and increases the serum concentration of NEFA (47), limits propionate production (58) and stimulates the synthesis of ketone bodies, mainly BHB (59, 60). In this study, cholesterol and BHB blood concentrations were within values previously reported for sheep (49, 52), but lower than values reported by Kaneko et al. (31) for BHB; however, as the grazing management did not affect their concentration on non-metabolically challenged adult sheep, we suggest that substantial changes of these energy metabolites are more likely to occur when imposing energy intake restrictions below maintenance (50), which was not the case of our study, as animals of both treatments put on weight (data not shown), as demonstrated by Schons et al. (9) in a similar experiment. Likewise, alkaline phosphatase can be reduced under feed intake restriction, as occurred with sheep at a high stocking rate (61). Nonetheless in our study, its decrease in RT animals was not significant. As with energy metabolites, it is perhaps necessary a severe feed restriction to affect its concentration.

As explained above, albumin is indicative of mid-term protein status (54), while BUN is of readily dietary protein intake (62). Both metabolites were above the superior limit of 30 g/dL and 20.7 mg/dL, respectively, reported by Kaneko et al. (31) for adult sheep. As these metabolites respond positively to dietary CP, its excessive intake by sheep of both treatments is evident. BUN concentration on RN and RT sheep are comparable to values reported on sheep grazing temperate pastures with 25% of CP [blood urea equivalent to 33.1 mg/dL of BUN; (63)]. High BUN may cause reproductive inefficiency in sheep at values around 14.6 mg/dL (64–66). Such inefficiencies have been noticed on temperate pasture-based dairy herds (67, 68). Moreover, high concentration of nitrogen in the rumen could lead to less efficient ATP-producing fermentation pathways (69), high energy expenditure due to ureagenesis in the liver (70) or boost the emission of nitrous oxide from manure. In this latter regard, despite Savian et al. (71) noticed greater nitrous oxide emissions from feces, these represented <1% of the CO_2_-eq emitted as enteric methane (g/ha/day), which was 61% lower in the RN, with respect RT grazing (18). To avoid inefficiency associated to excessive dietary CP, it becomes necessary to test optimal levels of energetic supplementation on animals grazing under the RN management and assess the trade-off between the emission of greenhouse gases via urine and the carbon stock in soil on RN paddocks, hypothesized to be enhanced due to greater herbage growth (9).

Low energy intake can activate the pituitary-adrenal axis, suppress animals' immune response and impair their welfare. Within leukocytes or immune cells, neutrophils participate in phagocytosis, produce reactive oxygen species (highly toxic for engulfed bacteria) and antibacterial enzymes (72), and lymphocytes confer cell-mediated immunity through immunoglobulins (73). As under long-term stress the blood N:L ratio increases (11) in response to greater levels of glucocorticoids (32, 74), this ratio is a good proxy of long-term stress (75). Sub-optimal feeding (76), monotonous diets (77) or delaying feeding (78), affect leukocytes formation and function (47). In this study, the 17% higher N:L ratio on RT sheep, support our hypothesis that the grazing strategy penalizing individual OM intake by 18% could impair animal welfare via depressing the immune response. This is also suggested from the greater NEFA concentration, as animals with high circulating NEFA can have their immune system suppressed (Ingvartsen and Moyes, 2012). A greater N:L ratio (52) or the expression of hepatic proteins involved in immune response and inflammatory cytokines (53), as indicators of welfare impairment were also observed on sheep under sub-optimal grazing conditions.

Overall, RN sheep exhibited a metabolic and hematological profile that could be associated to a better nutritional status and immune response to stress, thus to welfare. Nonetheless, it is advisable that given the subjective nature of the welfare concept, no single physiological measurement is conclusive and that complementary assessments over the whole grazing season (e.g., inflammatory responses, oxidative stress, behavioral responses to the grazing environment denoting ingestive comfort or the opposite), should be considered for a broader evaluation of the well-being of grazing animals.

## Conclusion

Pastoral systems must be evaluated not only by their productivity and environmental impact, but also by how much they promote animal welfare. This study shows that the low-intensity/high-frequency grazing strategy (RN) allowed sheep to compose their diet mostly from bites performed on the top stratum of plants, contrary to a traditional strategy (RT) of high-intensity/low-frequency grazing, forcing sheep to compose diets mostly from bites performed on top and grazed parts of plants. The biting behavior of sheep grazing vegetative Italian ryegrass pastures under the RN strategy allowed them to have a greater intake of a diet with better quality and a biochemical-hematological profile positively associated with nutrition and immune response to stress, which means that well-managed pastures (e.g., RN) could be a good strategy to promote welfare in grazing conditions.

## Data Availability Statement

The raw data supporting the conclusions of this article will be made available by the authors, without undue reservation.

## Ethics Statement

The animal study was reviewed and approved by Ethics Committee for the Use of Animals (CEUA) of the Federal University of Rio Grande do Sul (UFRGS; protocol 3571).

## Author Contributions

AZ: conceptualization, writing original draft, investigation, review, editing, and field data collection. AM: field data collection and review and editing. JS: conceptualization, field data collection, and review and editing. AS: conceptualization, field data collection, and review and editing. JR: data curation, formal analysis, and review and editing. JB: writing, review and editing. MB: field data collection and laboratory analysis. CB: formal analysis and review. LQ: review and editing and laboratory analysis. SV: resources and review. VD: writing, review and editing and resources. PdF: conceptualization, supervision, funding acquisition, review and editing, resources, and project administration. All authors contributed to the article and approved the submitted version.

## Conflict of Interest

The authors declare that the research was conducted in the absence of any commercial or financial relationships that could be construed as a potential conflict of interest.
