# Supplementary figures and images for: Low-Intensity, High-Frequency Grazing Positively Affects Defoliating Behavior, Nutrient Intake and Blood Indicators of Nutrition and Stress in Sheep
Source: Front Vet Sci. 2021 Jun 22;8:631820. doi: 10.3389/fvets.2021.631820 (PMC8255917; doi:10.3389/fvets.2021.631820)

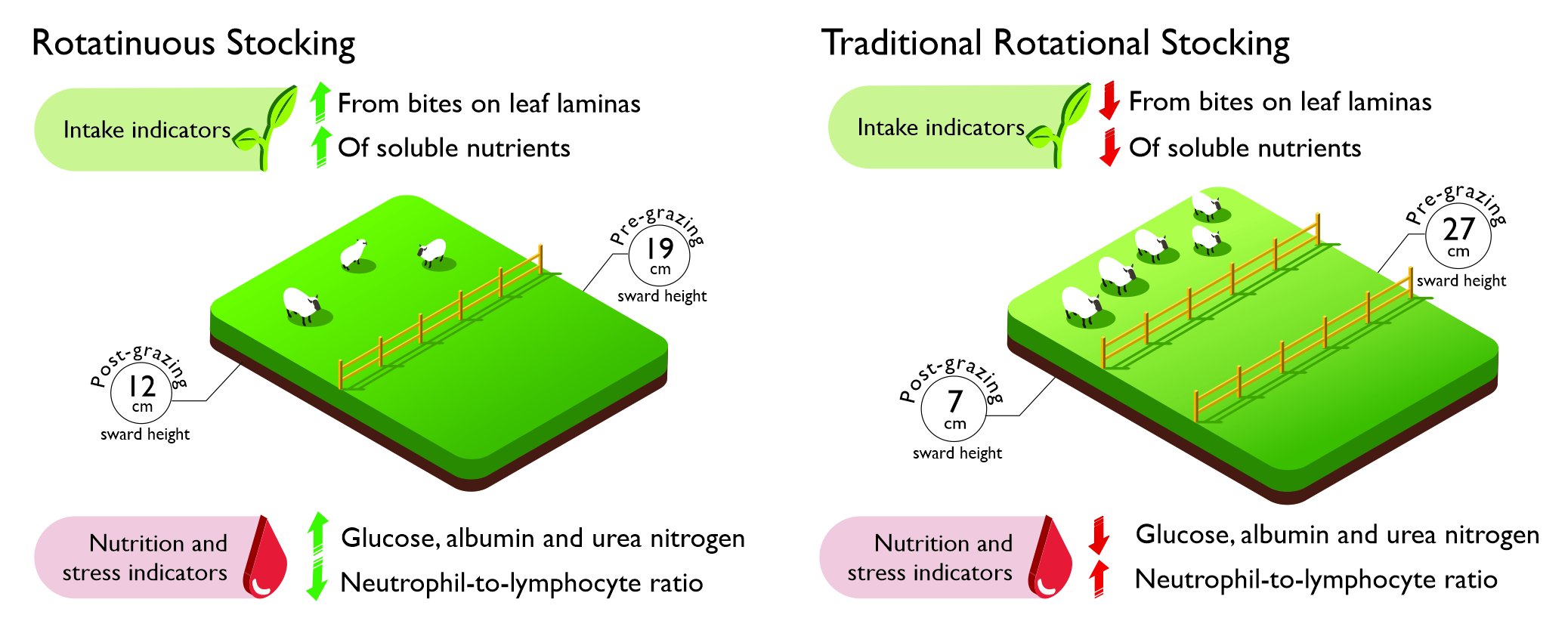

Supplement: Supplementary file 1 [file Image_1.TIF]
